# Splenic trauma from colonoscopy: A case series

**DOI:** 10.1016/j.ijscr.2020.04.057

**Published:** 2020-05-11

**Authors:** Dhaval D. Patel, Diane C. Shih-Della Penna, Shawn M. Terry

**Affiliations:** WellSpan York Hospital, 1001 S. George St., 2 Main, Surgical Services, York, PA 17403, United States

**Keywords:** Colonoscopy, Splenectomy, Interventional radiology, General surgery

## Abstract

**Introduction:**

Splenic trauma is quite rare after colonoscopy and can be overlooked as a complication when a patient presents with severe abdominal pain. It can be difficult to diagnose without appropriate imaging, but it should be considered as part of the differential in a patient arriving for evaluation of left upper quadrant abdominal pain.

**Presentation of case:**

In this case series, we discuss four patients who presented to our institution with splenic trauma specifically after colonoscopy. These patients were diagnosed with splenic trauma utilizing computed tomography (CT) scans of the abdomen and pelvis. They were all immediately transferred to our surgical intensive care unit (SICU) for close monitoring and serial hemoglobin checks. Two of the four patients had decreasing hemoglobin levels and were monitored until they underwent interventional radiology (IR) angiography and angioembolization. The other two patients had significant transfusion requirements and ultimately went to the operating room for an open splenectomy. All four of these patients did well after their interventions, although one of them required longer hospitalization while on the ventilator secondary to Haemophilus infection.

**Discussion:**

This case series recognizes that there is potential for quite severe splenic trauma after colonoscopy. While one of the four patients did have a history of prior splenic trauma, the other three had no history of trauma.

**Conclusion:**

These cases demonstrate that this complication should be managed similarly to traumatic splenic injury unrelated to colonoscopy, and that non-operative treatment remain a possibility. Certainly, non-operative management requires a SICU and IR capabilities to be successful. If the patient becomes unstable, they should undergo the appropriate operative intervention.

## Highlights

• There are 170 cases of post colonoscopy splenic injury reported in the literature.
• Management should be dictated by the patient’s clinical status and adhere to traumatic blunt splenic injury guidelines.
• Awareness of risk factors and post-procedure vigilance leads to prompt detection and intervention for this rare complication.

## 1 Introduction

Colonoscopic evaluations are performed for a variety of diagnostic and therapeutic reasons. Most complications occur during colonoscopies that have concurrent polypectomies [1,2]. Splenic injury due to colonoscopy is an exceedingly rare complication with only 170 recognized cases reported in the literature [1].

Colonoscopic-related splenic injury has been attributed to undue tension placed on the splenocolic ligament during scope passage [3]. Additionally, traction against existing peritoneal adhesions from prior splenic trauma or intra-abdominal infection may lead to capsular insult. Most patients often develop symptoms within the first 24 h [4].

We describe four cases of patients who experienced splenic trauma after colonoscopy, and who were treated by the Acute Care Surgery (ACS) team at a community teaching hospital. All cases initially presented at an outside hospital then transferred to our institution with surgical intensive care units (SICU) and interventional radiology (IR) capabilities. Two patients were treated with IR angioembolization with splenic preservation and two patients required open splenectomy.

## 2 Case report 1

The patient is a 62-year-old obese male with a history of prior colonoscopy-related microperforation and splenic injury, who was managed non-operatively during this previous episode. He underwent an elective outpatient surveillance colonoscopy and was found to have new rectosigmoid and cecal polyps. Polypectomy was performed and pathology ultimately demonstrated hyperplastic growth patterns. The patient recovered symptom free and was discharged home.

**Fig. 1:**
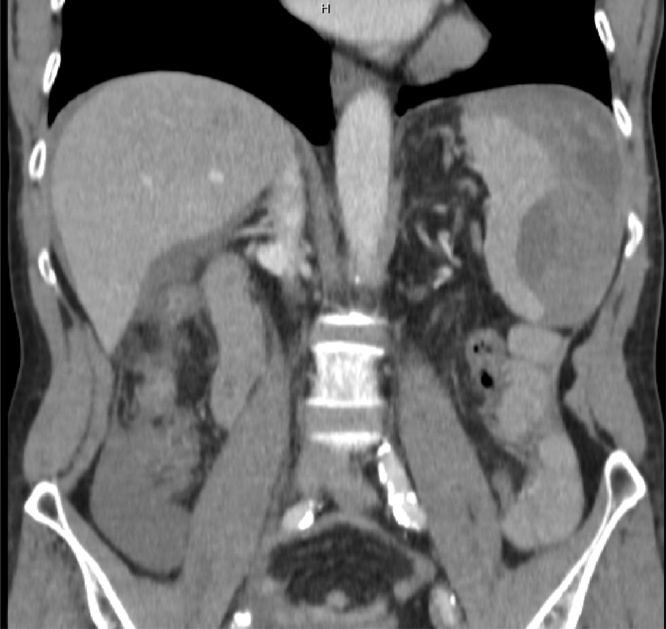
Perisplenic hematoma with no evidence of pneumoperitoneum.

Twenty-four hours later the patient presented to the emergency department complaining of severe left upper quadrant pain. He exhibited normal vital signs and had an initial hemoglobin of 11.7. A computerized tomography (CT) scan of the abdomen and pelvis with intravenous (IV) contrast demonstrated hemoperitoneum and splenic capsular rupture without contrast extravasation (Fig. 1).

The ACS team was consulted and elected to treat the patient non-operatively. He was admitted to the SICU where serial abdominal examinations and hemoglobin levels were performed. The patient’s pain improved and a clear diet was started.

**Fig. 2:**
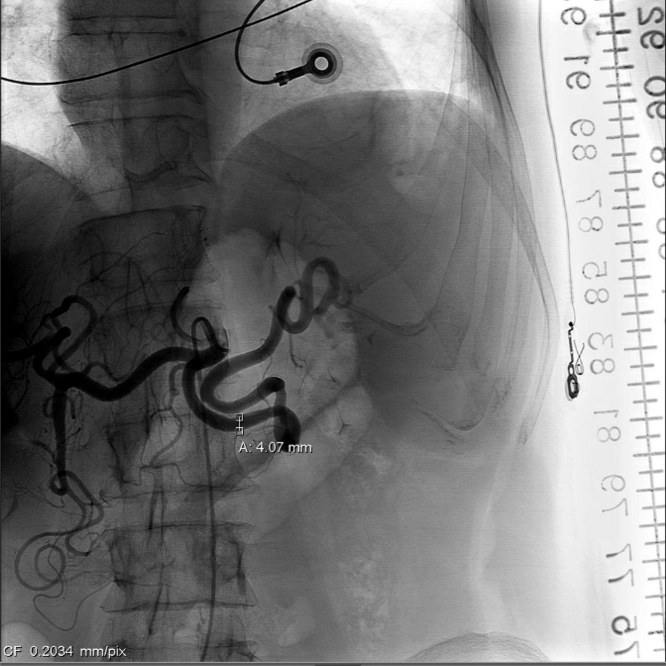
Angiogram after angioembolization and coiling demonstrating abrupt cessation of blood flow in the splenic artery.

Over the next 48 h, the patient’s hemoglobin slowly declined to 7.5 but he exhibited no signs of hypotension or peritonitis. He was taken to IR for angioembolization with a plan for splenic preservation. The proximal splenic artery was coiled (Fig. 2). The post procedure hemoglobin declined to 7.3 and the patient was transfused 1 unit of packed red blood cells (pRBC). The patient made an uneventful recovery and did not require any further transfusions. He was discharged home on hospital day 5 with a stable hemoglobin of 9.1.

## 3 Case report 2

This patient is a 47-year-old woman with a history of chronic abdominal pain who underwent an elective esophagogastroduodenoscopy (EGD) and a screening colonoscopy. On post-procedure day 7, she began having chest wall pain and pain with respirations. Her gastroenterologist requested that the patient obtain a chest x-ray (CXR) and an abdominal magnetic resonance imaging (MRI). The abdominal MRI demonstrated an encapsulated splenic hematoma and the CXR was unremarkable.

**Fig. 3:**
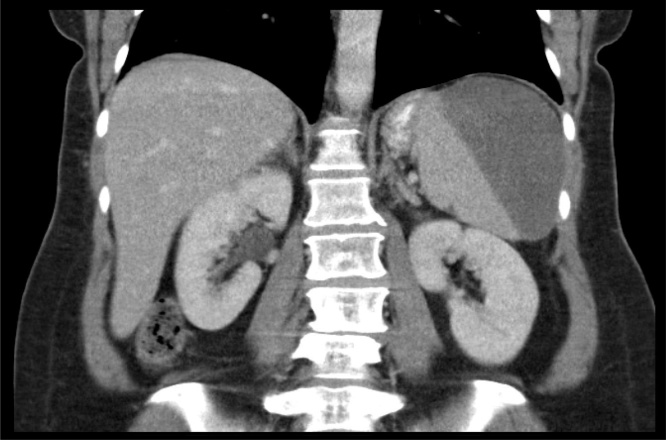
Large perisplenic hematoma.

The patient was initially monitored as an outpatient, but she experienced escalating left upper quadrant abdominal pain. She presented to an outside hospital on post-colonoscopy day 17, where a CT scan of the abdomen and pelvis with IV contrast demonstrated an increase in the size of the splenic hematoma, but no contrast extravasation (Fig. 3). She was transferred to our hospital and was admitted to the SICU by the ACS service.

**Fig. 4:**
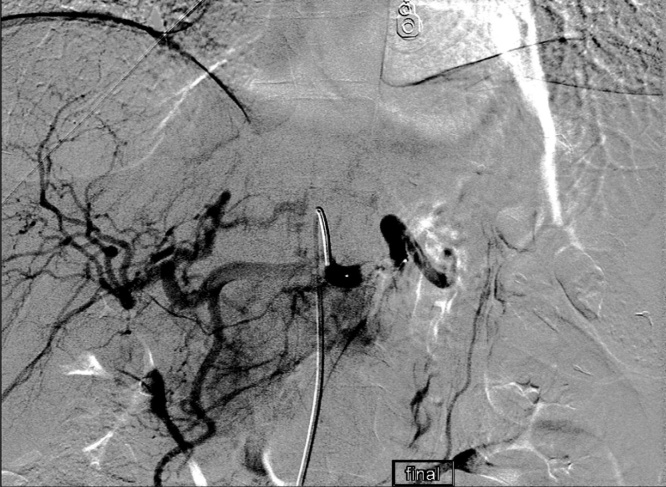
Angiogram after angioembolization and coiling demonstrating cessation of blood flow in proximal splenic artery.

On initial assessment, the patient demonstrated no evidence of systemic inflammatory response or hypovolemic shock with a presenting hemoglobin of 13.1. She remained stable with non-operative monitoring in the SICU with serial abdominal exams and hemoglobin levels. After reviewing the imaging and discussing her case with interventional radiology, she underwent elective proximal splenic artery angioembolization the following day (Fig. 4). Post embolization recovery was uneventful, and the patient did not require any transfusions. She was discharged home on hospital day 6 (post-colonoscopy day 23).

## 4 Case report 3

This patient is a 58-year-old female who underwent elective screening colonoscopy. She presented post-procedure day 1 to the same hospital with generalized abdominal pain. A CT scan of the abdomen and pelvis demonstrated a high-grade splenic laceration. She was admitted in stable condition to that facility for non-operative management. Over the first 24 h, progressive anemia resulted in transfusion of 6 units of pRBC and 6 units of platelets.

The patient developed post-transfusion respiratory distress and exhibited pulmonary edema on CXR. Her acute blood loss anemia persisted, and she was emergently transferred to our hospital on post-colonoscopy day 3. Due to the patient having exam findings of peritonitis and hypovolemic shock on arrival, she was taken emergently to the operating room (OR) for an open splenectomy. Intraoperatively, she was noted to have massive hemoperitoneum, splenic hilar avulsion, and active splenic artery bleeding. A splenectomy was performed and a Jackson-Pratt (JP) drain was placed in the left upper quadrant of the abdomen.

Her post-operative SICU course was complicated by continued respiratory failure and there were two failed attempts at extubation. An infectious workup demonstrated Haemophilus influenzae in her sputum and staphylococcus in her urine. Both infections were treated successfully with ceftriaxone. She was successfully extubated on post-operative day 10. The JP drain fluid analysis demonstrated minimal amylase levels and the drain was removed. The patient also experienced postoperative thrombocytosis and was started on aspirin 81 milligram daily. She continued to improve clinically and was discharged home on hospital day 13.

## 5 Case report 4

The patient is a 68-year-old female who underwent elective screening colonoscopy, which demonstrated internal hemorrhoids. After the procedure, she immediately experienced left upper quadrant abdominal pain. This was attributed to retained insufflated air and she was discharged home.

**Fig. 5:**
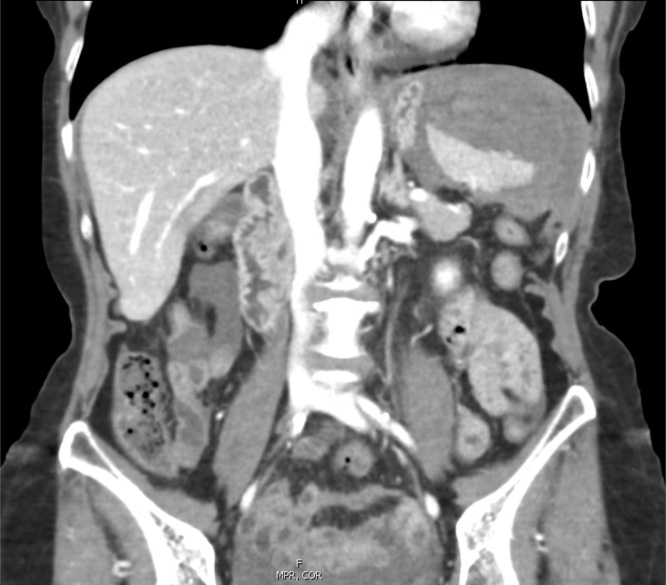
Large perisplenic hematoma with dense hemorrhagic ascites in the pelvis.

The patient’s pain increased and she was admitted to the hospital for evaluation. A CT scan of the abdomen and pelvis demonstrated moderate hemoperitoneum and a 1.9 cm splenic laceration with no active extravasation (Fig. 5). She was urgently transferred to our institution where she presented with tachycardia, normotension and an initial hemoglobin of 9.4.

She was admitted to the SICU for non-operative management. The patient suddenly complained of severe generalized abdominal pain and her repeat hemoglobin level was 6.9. She was taken to the OR for emergency open splenectomy with JP drain placement. Intra-operatively, she was transfused with 4 units of pRBCs. This patient’s splenectomy occurred approximately 14 h after colonoscopy. The patient was discharged home on hospital day 6 with a hemoglobin of 10.7.

## 6 Discussion

Colonoscopy is commonly performed by both gastroenterologists and general surgeons for diagnostic and therapeutic purposes. Most post-procedural complications are mild and self-limiting. Less common complications, such as splenic trauma, may go unrecognized and lead to significant morbidity. Persistent symptoms such as escalating left upper quadrant abdominal pain, tachycardia, and hypotension should raise suspicion for a splenic injury. Prior splenic trauma, history of intra-abdominal infection, or surgery increase this risk.

General treatment principles applied to this series of patients correlated with guidelines for management of traumatic blunt splenic injury. Unstable patients should be considered for immediate exploratory laparotomy and cell saver devices should be utilized if available and appropriate. Stable patients should have diagnostic imaging to confirm the diagnosis by a CT scan of the abdomen and pelvis with IV contrast. Active contrast extravasation mandates urgent IR angioembolization or open splenectomy. Patients that are hemodynamically stable with splenic injuries can be managed non-operatively. They should be admitted to a monitored intensive care unit for observation with orders for frequent checks of hemoglobin levels and serial abdominal exams. There should be a low threshold for intervention if symptoms worsen or acute blood loss anemia escalate. Summary data for the management of post-colonoscopy splenic injury patients is presented in Table 1.

**Table 1:** Summary data for the management of post-colonoscopy splenic injury patients.

Patient	Age	Gender	Anticoagulant/antiplatelet therapy	Prior abdominal surgery	Hgb prior to intervention	Intervention	Transfusion	Discharge hgb
1	62	M	none	none	11.7	IR embolization	1 pRBC	9.1
2	47	F	none	none	13.1	IR embolization	none	13.6
3	58	F	none	none	6.7	splenectomy	6 pRBC 6 platelets 2 FFP	9.8
4	68	F	none	none	9.4	splenectomy	4 pRBC	10.7

Two of the four patients in this case series fulfilled criteria for immediate exploratory laparotomy: hypovolemic shock in the presence of acute blood loss anemia and known splenic injury. In both cases, operative strategy mirrored a trauma exploratory laparotomy. We started with initial packing, anesthesia guided intra-operative resuscitation until hemodynamic stability was achieved, followed by open splenectomy. Prompt management of hemorrhage from the spleen can reduce the incidence of intra-operative hypothermia and coagulopathy. Utilizing cell saver technology can reduce the volume of allogenic red blood cell transfusions. Appropriate post-operative care should also include vaccines to prevent overwhelming post-splenectomy infection.

The other two patients were both managed non-operatively with successful splenic preservation. They were observed in the SICU and received catheter-directed IR angioembolization. Red blood cell transfusions were employed which were adherent to institutional blood bank clinical guidelines for symptomatic anemia. Splenic vaccines were not indicated for these patients.

While there are several known techniques to avoid colonoscopy-related injuries, no specific practice pattern can eliminate complications. Review papers have suggested that endoscopists become familiar with the associated risks: female sex, prior abdominal surgeries, anti-platelet or anti-coagulation medications, and concurrent polypectomy during colonoscopy [1]. Awareness of these associated risk factors and post-procedure vigilance can lead to prompt detection and timely intervention for this rare complication.

## 7 Conclusion

There are approximately 170 cases of post colonoscopy splenic injury reported in the literature. Successful treatment is dependent upon prompt recognition and intervention of this occurrence. Management should be dictated by the patient’s clinical status and adhere to traumatic blunt splenic injury guidelines. We recommend early transfer of these patients to a hospital with a SICU and IR capabilities. Finally, there should be strong consideration for in-hospital or adjacent facility colonoscopy procedures for patients identified as high risk for splenic injury.

## Declaration of Competing Interest

None.

## Funding

None.

## Ethical approval

IRB exempt.

## Consent

Written informed consent was obtained from the patients for publication of this case report and accompanying images. A copy of the written consent is available for review by the Editor-in-Chief of this journal on request.

## Author contribution

Dhaval Patel, MD: data collection, data analysis, writing of paper.

Diane Shih-Della Penna, MD: writing of paper, preparation of manuscript for publication.

Shawn Terry, MD: study concept, review of paper.

## Registration of research studies

Name of the registry: Research Registry.

Unique identifying number or registration ID: researchregistry5521.

https://www.researchregistry.com/browse-the-registry#home/.

## Guarantor

Dhaval Patel, MD.

## Provenance and peer review

Not commissioned, externally peer-reviewed. The research work has been reported in line with the PROCESS criteria [5].
